# Cacoëpy in Medicine*Read before the Buffalo Medical Club, Dec. 10th, 1879.

**Published:** 1880-02

**Authors:** J. W. Keene


THE BUFFALO

MEDICAL AND SURGICAL JOURNAL.

Vol. XIX.—FEBRUARY, 1880.—No. 7.

### Original Communications.

### CACOËPY IN MEDICINE.*

BY J. W. KEENE, M. D.

It is a matter of daily observation that physicians differ widely in their opinions. Differences in regard to pathology, diagnosis, prognosis and treatment, are so common that the variance is voiced in the saying that Doctors seldom agree. This difference is well exemplified in the pronunciation of our medical nomenclature. It seems that whatever variance may exist in matters of judgment, the pronunciation of medical terms, which is comparatively fixed and definite, should present a nearly perfect uniformity. This uniformity, however, does not exist,—except that in many cases the pronunciation is uniformly bad.

That the pronunciation of common English words is often incorrect, even among the educated, is well-known. Nor is this always the result of ignorance as to the proper pronunciation. In this, as in other matters, bad habits contracted in youth, cling to us with deadly pertinacity. The knowledge may not be in fault; but the vocal apparatus, once accustomed to produce a given sound, returns to it with all the fatal facility of error.

*Read before the Buffalo Medical Club, Dec. 10th, 1879.

The proposition just now made, that the correct pronunciation of medical terms is comparatively fixed and definite may, perhaps, be questioned; and with some justice it is admitted. Even in regard to English words of common occurrence our authorities differ, and often permit a choice between two or more very different ways of pronouncing the same word. We will admit this as one cause of our faults in orthoëpy, so far as it holds good. Again, it may be objected that the Latin, from which our medical nomenclature is so largely recruited, is in a sort of transition state, hanging poised, like the coffin of the Moslem prophet, midway between the terra firma of substantial English, and the dreamy heaven of Continental principles of orthoëpy. And it may be well to add that this is also true of the Greek; since, with very few exceptions, words of Greek derivation reach us in the Latin form. This transition has given rise to a barbarous jargon, resulting from the mingling of the two systems, and may well be admitted as a second and important cause of our bad pronunciation. But these two causes are sufficient to explain the grievous faultiness of our pronunciation only in a measure. Errors arising from these causes are natural and excusable ones; but a large percentage of all our errors in orthoëpy are inexcusable and unnatural, and are to be explained only by the perversity of the human mind. This last cause is the dominant one, and to it may be attributed that vast aggregate of errors in pronunciation, to a few of which reference will be made in this paper.

We have all been taught in our youth that the branch of the science of language which treats of the correct pronunciation of words is called orthoëpy, from the Greek *orthos*, right or correct, and *epos*, a word. The improper pronunciation of words will, from a similar derivation, be properly called cacoëpy, from *kakos*, bad or incorrect, and *epos*, a word. And lest the very title of our paper be made an example of that which it purposes to deprecate and correct, let it suffice to say that the accent falls on the first syllable cā′coëpy, analogous to or′thoëpy.

But some who insist on saying ortho′epy, or ortho-ē′py—and their name is legion—will undoubtedly call our title caco′epy or caco-ē′py in spite of the caution. "Such is human perversity." If the term be sought for in the dictionaries of the present, the search will be in vain; but unless we reform our pronunciation of medical nomenclature, cacoēpy will be an imperative necessity in the bright lexicon of the future.

Cacoēpy, then, is our theme. It is entirely foreign to our present purpose to discuss the propriety of employing the Continental method of pronunciation in our nomenclature, or to criticise those who employ it. So many of our physicians acquire their profession in the Continental schools of medicine, or at least add to their professional knowledge by studying there, that it is not practicable nor desirable to hold them amenable to English rules of orthoēpy. It would certainly be just and reasonable to expect of them a proximate uniformity, and a pronunciation of all words strictly and essentially Latin according to Continental methods. But even this we will not insist upon. So far as it is a question of English or Continental methods we will only ask that but one system be employed in the same word. And numerous examples may be noticed hereafter of a commingling of both in a single word. The aim is not to excite opposition, but to invite attention to that which is so outrageously bad that all will agree to the propriety of correcting it. The question then of the proper pronunciation of -itis as a terminal indicative of inflammation will not be discussed, except where the pronunciation of the remainder of the word is inconsistent. The word which our English dictionaries agree in pronouncing bronchī′tis* may with perfect propriety as a Latin word be called bronchī′tis; but it is highly inconsistent to pronounce meningī′tis, vaginī′tis, laryngī′tis, &c., meningī′tis, vaginī′tis, or laryngī′tis. If the termination is pronounced -ītis, according to the Continental system, the balance

---

*Fāte, făt, fāther—mēte, mĕt—pīne, pĭn, marīne—tōne, ŏn. Observe the accent marks.

of the word should conform to that system, and the pronuncia-tion becomes mā-nin-gï'tis, wä-gin-ï'tis and lar-yn-gï-tis, the g being hard as it always is in the Continental system. Thus consistency is not offended.

But a large number of physicians—it is safe to say the majority—do not consider that they are using the Continental method of pronouncing Latin when they say -ïtis, and are oblivious to the fact that they are employing Latin at all, save in form and derivation. They consider the words as English, and amenable to English rules of orthoëpy. Indeed, this outrage on a respectable language (and it is often doubtful whether it is the Latin or English that suffers) was habitually practiced by doctors long before the Continental method of pronouncing Latin was employed in a single public school or college in our land. But enough of this. Hundreds of examples like those cited above could readily be given, and many no doubt have already occurred to you; but time and disposition alike are wanting to present others. -Ïtis, or ītis, it is all the same;—only let us strive to be consistent in this as in all things.

Now let us turn our attention to cases where this plea of Continental method will not excuse mistakes. The examples are so numerous that one scarcely knows what to select from such an "embarras de richesse." Prīmĭp'ara, multĭp'ara, plurĭp'ara we have often heard called prīmipăr'a, multipăr'a, pluripăr'a, even by those whose hands, wonderfully endowed with the "tactus eruditus," have conferred upon the fair candidates the initial honor of maternity—the "first degree," that of primip'ara and often, too, the "higher degrees" of multip'ara, or plurip'ara. Impetī'go, prurī'go, porrī'go, &c., are wrested to impet'ĭgo, pru'rĭgo and por'rĭgo. Vertī'go as a Latin word follows the same analogy, but is Anglicised as ver'tigo or vertī'go. Gynecology (jin) is often pronounced with the initial g hard, probably to indicate a knowledge of its derivation from the Greek word, *gunee*, a woman, and *logos*, a treatise; but the same rule applies to both g's; if one is hard the other should be, if either is soft both are so.

The word so often called asthe′nia, is properly asthenī′a, both as an English and as a Latin word. A number of analogous words ending in -ia have the i long and accented, being derived from the Greek diphthong *ei.* Such are synechī′a, exangī′a, neurasthenī′a, eusthenī′a, enteropathī′a, energī′a. And, to prevent misunderstanding, it may be well to say that with the exception of these few words here cited to show analogy, every error mentioned in this paper has been heard by the writer, and many of them no doubt have been committed by him as well as by others. Rosē′ola, rubē′ola, arē′olar, alvē′olar, &c., smite the ear as roseō′la, rubeō′la, areō′lar, alveō′lar. Febric′ula becomes febricu′la; epu′lis, e′pulis; horde′olum, hordeo′lum. It may be claimed, and with some justice, that it makes little difference what the disease, or the drug exhibited, is called, if only the patient is cured. Certainly an en′ema will provoke stools equally copious if miscalled an enē′ma; the umbilī′cus is no more likely to become the seat of hernia for being mispronounced umbil′icus; diabetes mellī′tus will no doubt exhibit sugar in the urine just as certainly if called mel′litus; albu′men will be found in the urine of Bright's disease neither more nor less if it is called al′bumen; the abdō′men does not retract if called ab′domen; the massē′ter muscles do not refuse to masticate our food when called mas′seter; nor does the grim cadā′ver rise up in wrath to resent the ignominious title of cadăv′er. What difference then does the mere matter of pronunciation make? Certainly it serves to distinguish the learned from the unlettered, the educated man from the clown. When we hear the uneducated complain of "rheumatiz," "noorolergy" or "janders;" of "a catarrhical condition of the head," of "diarrhee," "dyspepshy" or "ulsters on the legs," it excites no astonishment; at most it provokes a smile and argues them unlearned. But greater accuracy is reasonably expected of members of a learned profession. A distinguished professor may use "labium majorum," instead of labium majus, as the singular number of labia majora, and yet not excite those sensitive parts; or speak of the

"cachexia" (ch as in chair) which renders the patient liable to a given affection, and not bring on the disease. A physician may recommend that the man dying of suspected violence should make an "anti-mortem" statement with strong probability that whatever statement the man may make, he would gladly make to that effect, and yet the grim Knight of the scythe may not be provoked to unseemly haste in claiming his victim. Another may speak of micrococci and echinococci (-coc′sī) as "micro-cock-eye" and "echino-cock-eye," and these tiny forms of life will not resent the implied imperfection of their ocular apparatus.

He may even call the father of medicine "Hip-po-crātes"—in three syllables, ignoring the e—and give no offense to us his unworthy children, whose regard for the paternal ancestor of our art may have become a trifle dulled by the lapse of years since the time when that venerable gentleman purged the ancient Greeks, and delivered their wives of babes destined to make the Grecian name and race illustrious through all time.

There is little doubt that ec′zema tests the skill of the dermatologist, quite as much when called eczē′ma; and he is entitled to equal credit for curing it under either name. Synechī′a taxes the resources of the oculist no less than when called synē′chia; nor is tinnī′tus aurium a more suggestive symptom to the aurist, or less annoying to the patient than when spoken of as tin′nitus aurium. Still if these diseases are ever cured it is because they are treated better than they are pronounced. Finally, a cicā′trix contracts as surely as a cicăt′rix; hamă′melis is just as inert as hamamē′lis; and verā′trum is no more active than veră′trum, or verăt′rum.

Let us glance for a moment at our drugs, and see what a woful medley their pronunciation presents. Quĭnīne′ for example—a most valuable drug in its place—has not only suffered abuse in its administration, being prescribed to-day by intelligent physicians to fulfil every possible and impossible indication in almost every disease that human flesh is heir to, but insult has been added to injury by "calling it names." Webster will have us call it quĭn-īne′ or quī′nīne; Worcester generously allows a choice of accent quĭn-īne′ or quĭn′īne. Both agree on quĭn-īne′, surely sufficient authority to establish a pronunciation. It would seem that these three authorized pronunciations might give sufficient latitude for choice even to the most fastidious. But beside these we hear of "quī-nīne′," "quĭn′ïne," "quin-ïne′," "quī′nïne," and "kineen," and so on, to the verge of the impossible, and even beyond.

Our druggists have much to answer for in many ways; and in this matter of cacoëpy they stand preëminent as the champion cacoëpists. But while they fill our prescriptions with absolute accuracy, giving us what the prescription calls for—no more and no less—with drugs of good quality, we will overlook the stupendous perversity of the craft in calling gum tragacanth, gum "trajacanth," contrary to every principle of pronunciation in any tongue. We will ignore such mongrel terms as "liquor (licker) ammoniae acetä′tis," and be silent when they speak of "podoph′-ylline," and "copee′ba." When they sell us "verä′trum vi′ride," we will not insist on either the English verā′trum viride, or the Continental wā-rä′troom wee′ree-dā, so long as the article is satisfactory in its action, but will allow them the privilege of mixing the two systems as they mix their drugs.

Nor would it be necessary to insist that every physician should pronounce bubonocele, cystocele and hematocele, bubon-ocē′le, cystocē′le, hematocē′le with the added syllable as the Latin requires. They are Latin words to be sure, and no such English words are recognized by our lexicographers. Yet we have bronchocele, hydrocele and varicocele Anglicised, and may take the liberty of following analogy. Nor will we insist on pletho′ra or trache′a, as the Latin demands, since both are Anglicised—pleth′ora or pletho′ra, tra′chea or trache′a. We would not have our physicians become pedantic in this or in any direction; but there is ample room for reform far removed from suspicion of pedantry.

There now remains a large number of words essentially English in form, whose pronunciation is often incorrect. A few of these—and only such as are common medical terms—are added. The first pronunciation given being the correct one, the second the common error:

| | |
|---|---|
| apparā′tus | apparä′tus |
| cer′vical | cervī′cal |
| adult′ | ad′ult |
| af′ferent | affer′ent |
| allop′athy | allopath′y |
| cav′ernous | cavern′ous |
| edĕm′atous | edē′matous |
| parī′etal | pariē′tal |
| afflā′tus | afflä′tus |
| canīne′ | cā′nīne |
| dys′entery( dis-) | dys′entery (diz) |
| cŏch′ineal | cō′chineal |
| salīne′ | salïne′ |
| aryt′enoid | aryten′oid |
| fē′brīle or fĕb′rīle | fē′brīle |
| sē′nīle | sĕn′īle |
| acclī′mate | ac′climate |
| flac′cid (flack′sid) | flaccid (flas′sid) |
| mĕt′ric | mē′tric |
| gā′seous (gaz) | găs′eous (gas) |
| lethar′gic | leth′argic |
| sin′ew (you) | sin′ew(sin′oo) |
| hȳ′datid or hy̆d′atid | hydăt′id. |
| mŏl′ecule | mō′lecule |
| im′potent | impo′tent |
| ephĕm′eral | ephē′meral |
| calor′ic | căl′oric |
| obĕs′ity | obē′sity |
| erysip′elas | erysip′elas(ir) |
| ener′vate | en′ervate |
| mĕd′ullary | medul′lary |
| diphthē′ria(dif) | dipththēria (dip) |
| syrup (sī′rup) | syr′up(sir′rup) |
| jăl′ap | jal′ap (jol) |
| morph′īne | morph-ïne′ |
| i′odīne | i′odïne |
| brŏ′mīde | brō′mīde |
| ox′īde | ox′īde, |

and a large number of words, chiefly chemical terms, ending in -ine and -ide, in most of which the i is short, but may be long in some out of deference to long established custom. There is no authority for -ïne.

Now is it not unworthy of men who are in some sense teachers of mankind, to be thus negligent and indifferent in regard to the pronunciation of the technology of their profession? It may be said that this is a trifle. The story is told of Michael Angelo that a friend visited him when he was finishing a statue. Some time after he came again and found the sculptor still at the work. Reproached for his apparent idleness the sculptor exclaimed, " Not so, I have retouched this part and polished that; softened this feature and brought out that muscle. I have given this lip more expression, and that limb more energy." " Well, these are but trifles after all," said the friend. " Possibly," replied Angelo, " but remember that trifles make perfection, and perfection is no trifle."

But this is no trifle. As a profession we depart widely from rules of orthoëpy in our nomenclature; and for an educated man, a member of a learned profession, to make the blunders we have pointed out, is infinitely worse than for the uneducated to say "noorolergy," "rheumatiz," or "diarrhee." The fact is, our teachers in medical colleges are in a great measure responsible. We take our pronunciation from them unquestioningly, as we too often receive their other teachings. The remedy is evident. Care on the part of instructors to teach nothing wrong, even in pronunciation, and a spirit of investigation in the student, which will lead him to accept nothing merely because his professor tells him so, but to look into all matters for himself, and follow none but reliable and established authorities.

